# Ferredoxin Competes with Bacterial Frataxin in Binding to the Desulfurase IscS*[Fn FN2]

**DOI:** 10.1074/jbc.M113.480327

**Published:** 2013-07-09

**Authors:** Robert Yan, Petr V. Konarev, Clara Iannuzzi, Salvatore Adinolfi, Béatrice Roche, Geoff Kelly, Léa Simon, Stephen R. Martin, Béatrice Py, Frédéric Barras, Dmitri I. Svergun, Annalisa Pastore

**Affiliations:** From the ‡ MRC National Institute for Medical Research, The Ridgeway, London NW7 1AA, United Kingdom,; the § European Molecular Biology Laboratory, EMBL c/o DESY, Notkestrasse 85, Hamburg D-22603, Germany, and; the ¶ Aix-Marseille Université and; ‖ Laboratoire de Chimie Bactérienne, Institut de Microbiologie de la Méditerranée, UMR 7283, CNRS, 31 Chemin Joseph Aiguier, 13009 Marseille, France

**Keywords:** Biophysics, Computer Modeling, Iron Metabolism, Iron-Sulfur Protein, Nuclear Magnetic Resonance, Protein Structure

## Abstract

The bacterial iron-sulfur cluster (*isc*) operon is an essential machine that is highly conserved from bacteria to primates and responsible for iron-sulfur cluster biogenesis. Among its components are the genes for the desulfurase IscS that provides sulfur for cluster formation, and a specialized ferredoxin (Fdx) whose role is still unknown. Preliminary evidence suggests that IscS and Fdx interact but nothing is known about the binding site and the role of the interaction. Here, we have characterized the interaction using a combination of biophysical tools and mutagenesis. By modeling the Fdx·IscS complex based on experimental restraints we show that Fdx competes for the binding site of CyaY, the bacterial ortholog of frataxin and sits in a cavity close to the enzyme active site. By *in vivo* mutagenesis in bacteria we prove the importance of the surface of interaction for cluster formation. Our data provide the first structural insights into the role of Fdx in cluster assembly.

## Teaser

**Background:** The bacterial *Isc* operon contains a ferredoxin whose precise role is unknown and a desulfurase enzyme.

**Results:** We have structurally characterized the complex of *Escherichia coli* ferredoxin with the desulfurase IscS.

**Conclusion:** We show that ferredoxin occupies a groove close to the active site.

**Significance:** Our results shed light into the mechanism of iron-sulfur cluster biogenesis.

## Introduction

Iron-sulfur (Fe-S) clusters are essential prosthetic groups that provide an important source of redox potential to the cell. They are usually coordinated to proteins by cysteines and histidines, sometimes complemented by aspartic groups. Fe-S cluster proteins are ubiquitous and perform a variety of roles including electron transfer, enzyme regulation, and regulation of gene expression (1, 2). Because both iron and sulfur are toxic to the cell, assembly and repair of Fe-S clusters has to be tightly regulated. Specific metabolic machines have evolved for this purpose. They are highly conserved between eukaryotes and prokaryotes and in the latter are encoded in specific operons implying a specific involvement in Fe-S cluster assembly. Any disruption/mysfunction of this regulation results in disease as is the case with the neurodegenerative Friedreich ataxia, which is caused by reduced levels of frataxin, an iron-binding protein that regulates Fe-S cluster assembly (3, 4).

**SCHEME 1:**
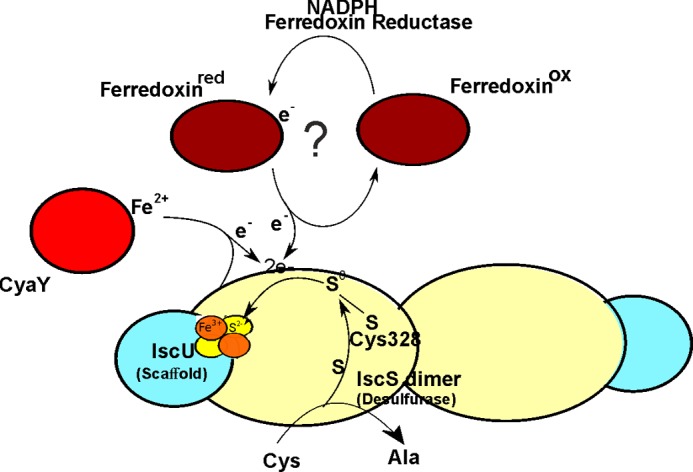


The main players of the Fe-S cluster assembly machine are a pyridoxal phosphate-dependent desulfurase and a Fe-S cluster scaffold protein (designated IscS/Nfs1 and IscU/Isu in bacteria and in eukaryotes, respectively) (5–8). They form a heterotetramer. The desulfurase converts l-cysteine to l-alanine, and S^0^, forming a highly reactive persulfide on the catalytic cysteine. S^0^ is subsequently transferred as S^2−^ to the scaffold protein, and along with Fe^3+^, is coordinated by the thiolate ligands of three highly conserved cysteine residues and forms a [2Fe-2S] cluster (Scheme 1). Generation of [4Fe-4S] clusters occurs through reductive coupling of two [2Fe-2S] clusters in holo-Isu/IscU homodimers (9). Along with IscS and IscU, other components are part of the machine. Among these is a ferredoxin (Fdx),^2^ which seems to have an important albeit unclear role. It has in fact been shown that genetic disruption of the endogenous *Fdx* gene both in prokaryotes and eukaryotes retards the activities of Fe-S cluster containing enzymes (10–13). In Fdx-depleted yeast and HeLa cells, electron paramagnetic resonance (EPR) and Mössbauer analyses show the absence of Fe-S clusters and the presence of aggregated Fe^3+^ nanoparticles in mitochondria (10–12).

A way to obtain further information on the functional role of Fdx is to characterize its interactions with the other components of the Fe-S cluster assembly pathway, a knowledge still limited: *Escherichia coli* Fdx was shown to interact with IscS, HscA, and IscA by co-purification (14, 15); IscS, in the presence of l-cysteine, was shown to be capable of repairing nitric oxide-modified Fdx [2Fe-2S] clusters (16). Cluster-free (apo) Fdx was shown to be an acceptor for HscA·HscB-mediated transfer of [2Fe-2S] clusters from IscU (17). Cluster-loaded (holo) Fdx was also shown to be active in the reductive coupling of two [2Fe-2S]^2+^ clusters to form a single [4Fe-4S]^2+^ cluster on homodimeric IscU (9).

To gain new insights into the role of holo-Fdx in Fe-S cluster assembly we have characterized the structure of the *E. coli* holo-Fdx·IscS complex using a combination of nuclear magnetic resonance (NMR), mutagenesis, and small angle x-ray scattering (SAXS). We also tested the interaction in the presence of IscU and the bacterial ortholog of frataxin, CyaY, whose complexes with IscS have been previously characterized (18–20). We found that holo-Fdx and CyaY compete for the same binding surface on IscS, raising the question of whether they play antagonizing roles in regulating IscS function. We validated our modeled structure *in vivo* by mutagenesis studies in *E. coli*.

## EXPERIMENTAL PROCEDURES

### 

#### 

##### Protein Production

The DNA of *E. coli fdx* was amplified by PCR from *E. coli* genomic DNA with a 5′ NcoI restriction site and a 3′ stop codon and NotI restriction site and cloned into a modified pET-24 vector (EMBL Hamburg) with a GST tag, His tag, tobacco etch virus protease cleavage site and NcoI restriction site in tandem upstream of the NotI restriction site. IscS mutants were prepared by site-directed mutagenesis of the construct pET-11 IscS (EMBL-Hamburg) (21) using QuikChange® (Stratagene).

After cell grown, induction, and lysis, Fdx and its mutants were purified by affinity chromatography using nickel-nitrilotriacetic acid-agarose gel (Qiagen). His-tagged tobacco etch virus protease (in-house) was used to cleave the N-terminal GST/His tag under dialysis overnight at 4 °C. The reaction mixture was passed through a nickel-nitrilotriacetic acid-agarose gel and further purified using a 16/60 Superdex G75 column (GE Healthcare) followed by ion exchange using a MonoQ HR 5/5 column (Pharmacia Biotech). Protein concentration was determined using ϵ_280_ = 6,990 m^−1^ cm^−1^. The amount of holo- with respect to apo-Fdx in the sample was estimated by taking the *A*_458_/*A*_280_ value of >0.45 to be >90% holo-Fdx (22).

Unlabeled *E. coli* CyaY, IscU, IscS, and its mutants were expressed and purified as described previously (19, 23–26). Singly and doubly ^15^N- and ^15^N/^13^C-labeled proteins were expressed in *E. coli* BL21(DE3) in M9 minimal medium prepared with [^15^N](NH_4_)_2_SO_4_ (^15^N_2_, 99%, CIL) and/or d-[^13^C]glucose (U-^13^C_6_, 99%, CIL). For [^15^N/^2^H]Fdx the growth medium was prepared with 99% deuterium oxide (D, 99.9%, CIL) and d-[^12^C/^2^H]glucose (1,2,3,4,5,6,6-D7, 98%, CIL).

##### NMR Titrations

Full spectral assignment was obtained by standard methods and deposited to the BMRB database (entry number 19273). [^15^N]- or [^15^N/^2^H]Fdx or Fdx mutants were titrated with IscS, IscS mutants, IscU and/or CyaY, all in NMR binding buffer (20 mm Tris, 150 mm NaCl, 20 mm tris(2-carboxyethyl)phosphine, pH 8) at 298 K. ^15^N-SOFAST HMQC spectra (27) were recorded on Bruker 700 AvanceIII and Bruker 600 MHz AvanceI spectrometers with TCI Cryoprobes.

##### Model Building and Validation

Protein docking between IscS and Fdx was computed on the HADDOCK server (28) using PDB 1P3W and 1I7H as starting structures, respectively. For IscS active ambiguous interaction restraints (AIRs) were defined as Arg^220^, Arg^223^, and Arg^225^ for the first protomer and Arg^112^ and Arg^116^ for the second promoter and passive AIRs were defined automatically. For holo-Fdx, active AIRs were defined as Asp^70^, Asp^71^, Asp^74^, Glu^80^, and Glu^82^ and passive AIRs were defined automatically by HADDOCK. The model coordinates are available as supplemental data.

##### Biolayer Interferometry (BLI)

All experiments were performed in 20 mm HEPES, pH 7.5, 150 mm NaCl, 2 mm tris(2-carboxyethyl)phosphine, and 0.5 mg/ml of bovine serum albumin on an Octet Red instrument (ForteBio) operating at 25 °C. Streptavidin-coated biosensors with immobilized biotinylated holo-Fdx were exposed to different concentrations of IscS (0–60 μm) in the presence and absence of 200 μm IscU. In the competition assay, streptavidin-coated biosensors with immobilized biotinylated holo-Fdx were exposed to 10 μm IscS at different concentrations of CyaY (0–100 μm), in the presence and absence of 30 μm IscU. The *K_d_* values were obtained by analyzing the BLI amplitude as a function of the titrand concentration.

##### SAXS Methods

Synchrotron radiation x-ray scattering data were collected on the EMBL P12 beamline at the PETRA III storage ring (DESY, Hamburg). Measurements were carried out at 10 °C with 2.5–5.0 mg/ml of solutions. The data were recorded using a 2-m PILATUS detector (DECTRIS, Switzerland) at a sample detector distance of 3.0 m and a wavelength of λ = 0.1 nm, covering the range of momentum transfer 0.12 < *s* < 4.50 nm^−1^ (*s* = 4π sinθ/λ, where 2θ is the scattering angle). No measurable radiation damage was detected. Data treatment was carried out by ATSAS package (29) according to a protocol already described (19). The scattering patterns of the IscS dimer (PDB code 1P3W) and IscS·Fdx complex were calculated using CRYSOL (30).

##### In Vivo Validation

The strains used in this work are *E. coli* DV901 derivatives (MG1655 *lacIpoZ*Δ(*Mlu*) P*_iscR_*::*lacZ*) (31). The Δ*fdx*::kan KEIO mutation was introduced into DV901 by P1 transduction and confirmed by PCR. Strains were grown in Luria-Bertani (LB)-rich medium at 37 °C under aerobiosis. When required, kanamycin and ampicillin were used at 25 and 50 μg/ml, respectively.

To construct the pFdxWT plasmid, the coding region of *fdx* was amplified from genomic DNA from the *E. coli* MG1655 strain by PCR using primers for NcoI-FdxUP/HindIII-FdxDO (NcoI-FdxUP, 5′-CCGGCCATGGCACCAAAGATTGTTATTTTGCCTCAT-3′; HindIII-FdxDO, 5′-CCGGAAGCTTTTAATGCTCACGCGCATGGTTGATAGTGTA-3′. The *fdx* product was digested and ligated in NcoI/HindIII-linearized pBAD24 vector. The pFdxD70K and pFdxD70KD74K plasmids, containing either an Asp to Lys^70^ mutation or Asp to Lys^70^ and Lys^74^ mutations, were constructed as follows. The coding region of each mutated version was amplified from the pET24-GSTfdxD70K and pET24-GSTfdxD70KD74K vectors using primers NcoI-FdxUP/HindIII-FdxDO. The two PCR products were digested with NcoI/HindIII and next subcloned into the pBAD vector.

For the β-galactosidase assay, strains were grown aerobically to an *A*_600_ ∼1.5 at 37 °C in LB-rich medium supplemented with arabinose (0.2%). β-Galactosidase assays were carried out as previously described (32).

## RESULTS

### 

#### 

##### Holo- but Not Apo-Fdx Binds Iscs and Competes for the CyaY Binding Site

**FIGURE 1.**
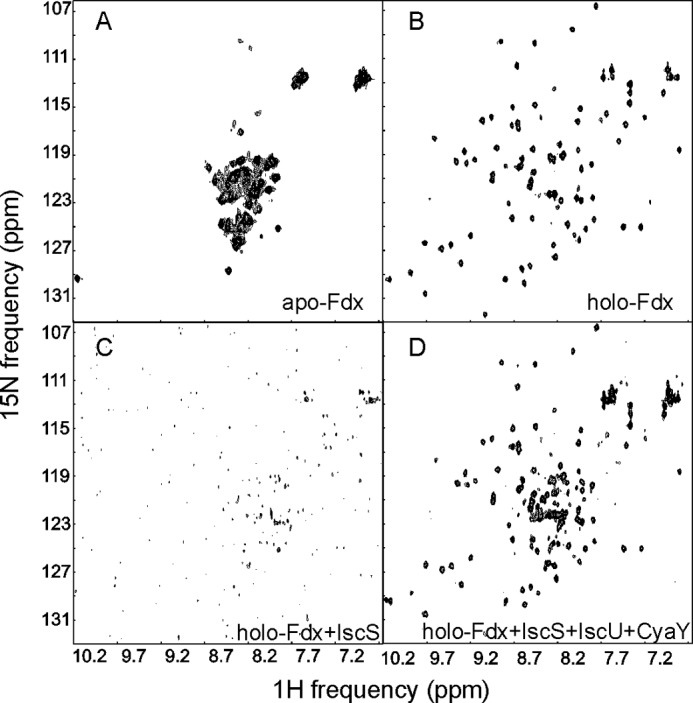
**Characterization of Fdx by NMR.** *A*, SOFAST HMQC spectrum of apo-Fdx. *B,* spectrum of holo-Fdx. *C,* spectrum of holo-Fdx titrated with a 0.3 molar ratio of IscS. Addition of IscU does not alter the spectrum (data not shown). *D,* spectrum of the same sample further titrated with 3 molar ratios of CyaY. The spectra were recorded at 700 MHz and 298 K.

After optimizing the holo-Fdx sample to ensure maximal occupancy of the cluster, we tested binding of the protein to IscS. We used ^1^H,^15^N-labeled SOFAST HMQC experiments recorded on labeled Fdx titrated with unlabeled IscS. The spectrum of apo-Fdx has the features of an unfolded protein and remained invariant during titration (Fig. 1*A*). At variance, the spectrum of holo-Fdx is typical of a folded protein and disappears upon titration with IscS (Fig. 1*B*). During the titration, few resonances of the holo-Fdx spectrum remain observable by 0.5 molar eq of IscS to holo-Fdx (Fig. 1*C*). This effect is likely to be caused by broadening of the holo-Fdx signals as a consequence of complex formation with the IscS dimer, which assuming a 1:1 complex, would lead to an overall molecular mass of 115 kDa. Complexes of this size are not usually observable without deuteration due to their large correlation times (τ_c_).

We then tested if Fdx could interfere with the binding site on IscS of either IscU or CyaY: the former packs against a hydrophobic surface around IscS residues Met^315^, Leu^383^, and Pro^385^, the latter inserts into a cavity formed between the active site and the dimer interface (19, 20). We successively added IscU and CyaY to the holo-Fdx·IscS sample. When we titrated the sample with IscU up to 1 molar eq of IscU to holo-Fdx, the signals of holo-Fdx remained unobservable indicating that IscU does not displace holo-Fdx from IscS (data not shown). When we added CyaY up to 3 molar excess over holo-Fdx, the spectrum of holo-Fdx reappeared, resembling that of free holo-Fdx (Fig. 1*D*). CyaY is thus able to displace holo-Fdx from IscS. These results tell us that the IscS interaction is specific for holo-Fdx in agreement with the indications of previous two-hybrid data (15) and that the binding site overlaps with CyaY.

##### Holo-Fdx Binds IscS with Micromolar Affinity

**FIGURE 2.**
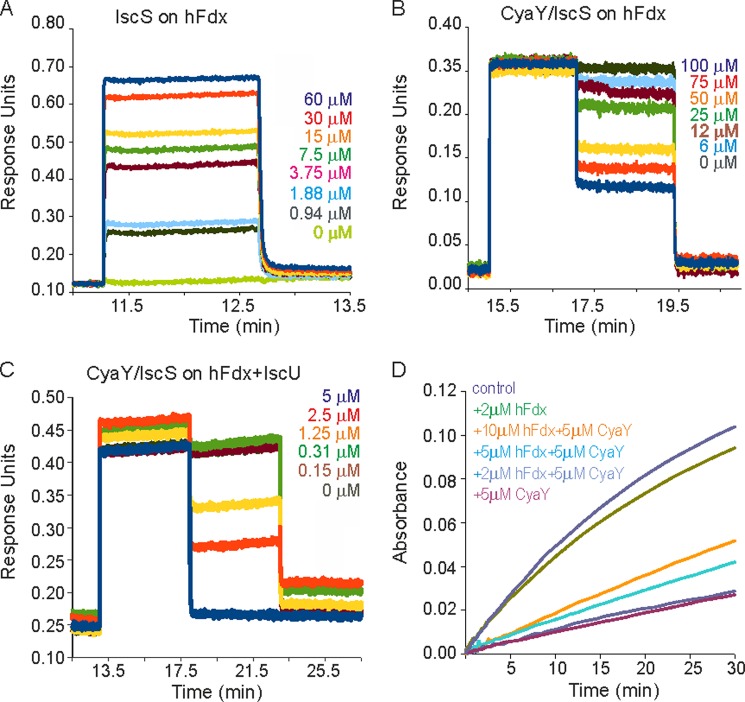
**Quantification of the affinity of IscS·Fdx interaction and effect of Fdx on Fe-S cluster assembly.** *A,* BLI profiles for IscS (at the concentrations indicated) binding to immobilized holo-Fdx. *B,* BLI profiles showing the displacement of IscS from immobilized holo-Fdx by CyaY (at the concentrations indicated) in the absence of IscU. *C,* as in *B* but in the presence of IscU. *D,* enzymatic Fe-S cluster reconstitution assay on IscU. Cluster assembly was followed by measuring *A*_456_ with time. From top to bottom: control with IscS and IscU only (*navy*), adding 2 μm holo-FDX (*green*), adding 5 μm CyaY and 10 μm holo-Fdx (*orange*), adding 5 μm CyaY and 5 μm holo-Fdx (*cyan*), adding 5 μm CyaY and 2 μm holo-Fdx (*blue*), and adding 5 μm CyaY (*magenta*).

To confirm and quantify this observation, we measured the affinity of holo-Fdx for IscS by BLI. We immobilized holo-Fdx and measured the binding affinity by titration of the protein with increasing concentrations of IscS (Fig. 2*A*). Holo-Fdx binds to IscS with fast *k*_on_ and *k*_off_ rates, which allow evaluation of the *K_d_* at the equilibrium. The *K_d_* value obtained was 1.5 ± 0.4 μm, which is comparable with the *K_d_* between IscU and IscS (1.5 ± 0.3 μm) (19).

We then checked holo-Fdx binding to IscS in the presence of IscU or CyaY using BLI. IscU has no effect on the affinity of holo-Fdx for IscS also indicating that there is no cooperativity for IscS binding (data not shown). This is in contrast to the cooperativity observed between IscU and CyaY in the CyaY·IscS·IscU complex (19). CyaY displaces IscS from the immobilized holo-Fdx (Fig. 2*B*). The calculated *K_d_* matches the value for the CyaY·IscS complex (23 ± 3 μm) (19) in agreement with a displacement of CyaY by holo-Fdx from the same binding site on IscS. When we repeated the competition assay in the presence of IscU (Fig. 2*C*), the *K_d_* value obtained from the displacement of IscS from holo-Fdx by CyaY matched the *K_d_* value for CyaY in the CyaY·IscS·IscU complex (35 ± 6 nm) (19) confirming no cooperativity. These results indicate that the binding site for holo-Fdx on IscS overlaps with CyaY and that holo-Fdx binding to IscS does not influence the affinity of the IscU·IscS complex.

##### The Effect of Holo-Fdx on Enzymatic Fe-S Cluster Formation on IscU

We wondered whether holo-Fdx and CyaY could have antagonizing roles in Fe-S cluster reconstitution. In the absence of holo-Fdx, CyaY inhibits Fe-S cluster assembly by IscS on IscU as described previously (3) (Fig. 2*D*). This inhibitory effect was attenuated with increasing concentrations of holo-Fdx in agreement with a competition between holo-Fdx and CyaY for IscS binding. In the absence of CyaY, holo-Fdx has a negligible effect on the rate of Fe-S cluster reconstitution. This result confirms that holo-Fdx competes with CyaY and attenuates the inhibitory effect of this protein on Fe-S cluster assembly.

##### Characterization of Holo-Fdx Surface of Interaction with IscS

**FIGURE 3.**
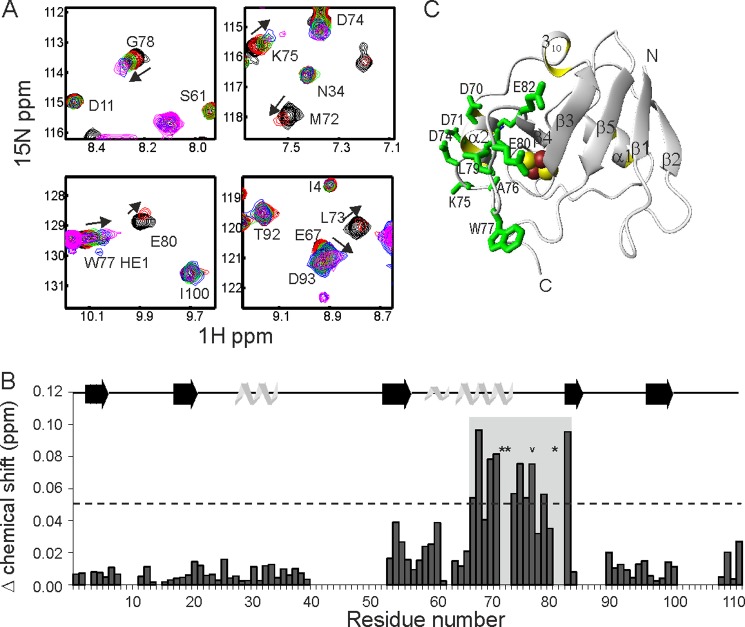
**Chemical shift mapping of the holo-Fdx surface interacting with IscS.** *A,* representative examples of the chemical shift perturbation observed in ^1^H,^15^N-SOFAST HMQC spectra of ^15^N/^2^H-labeled holo-Fdx upon titration with IscS to 0 (*black*), 0.1 (*red*), 0.2 (*green*), 0.3 (*blue*), and 0.4 equivalents (*magenta*). *B,* the Δ chemical shift at 0.3 equivalents of IscS per holo-Fdx residue. Residues denoted by a *star* are broadened at 0.3 equivalents. The indole N^ϵ1^H^ϵ1^ of Trp^77^ is denoted with an *chevron. C,* mapping the interaction on the holo-Fdx surface. The side chains exhibiting the largest Δ chemical shifts as denoted by the *shaded rectangle* in *B* are explicitly shown in *green*.

We mapped the surface of holo-Fdx interacting with IscS by NMR exploiting the chemical shift perturbation observed in the spectrum of holo-Fdx upon titration with IscS. Holo-Fdx was deuterated to attenuate the contribution of dipole-dipole interactions on the transverse relaxation. Chemical shift perturbations were readily observed up to 0.4 eq of IscS for Ile^54^, Val^55^, Gln^68^, Glu^69^, Asp^70^, Asp^71^, Met^72^, Leu^73^, Asp^74^, Lys^75^, Ala^76^, Trp^77^, Gly^78^, Leu^79^, Glu^80^, Glu^82^ and for the N^ϵ1^H^ϵ1^ indole group of Trp^77^ (Fig. 3*A*). Met^72^, Leu^73^, and Glu^80^ were completely broadened by 0.3 eq. At 0.5 eq and above the signals of holo-Fdx broadened. The largest variations of chemical shifts were observed for Gln^68^–Glu^82^ (Fig. 3*B*), suggesting that this region of holo-Fdx interacts with IscS directly. These residues are in helix 2 and the following loop of holo-Fdx (Fig. 3*C*) and form an exposed acidic patch on the surface of holo-Fdx.

##### Characterization of IscS Surface of Interaction with Holo-Fdx

**FIGURE 4.**
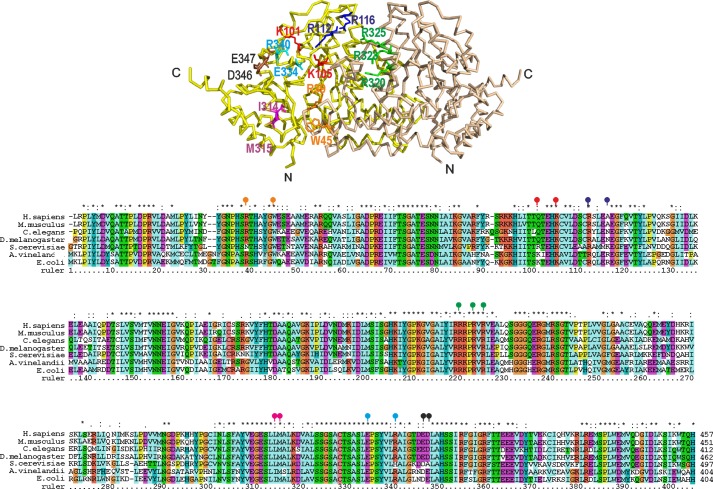
**IscS mutants designed to test interaction with Fdx and their positions in an IscS alignment.** *Top*, IscS backbone structure with the side chains of residues mutated indicated explicitly: R39E/W45E (*orange*), K101E/K105E (*red*), R112E/R116E (*blue*), R220E/R223E/R225E (*green*), I314E/M315E (*magenta*), E334S/R340E (*cyan*), D346K/E347K (*black*). *Bottom*, sequence alignment of representative species from bacteria to primates. The signal peptides are omitted in the eukaryotic sequences that start with the homology to the prokaryotic orthologues. The mutated positions are indicated with *balloons in the same color* coding as used in the structure.

To further characterize the surface of IscS interacting with holo-Fdx we tested the ability of *ad hoc* designed IscS mutants to bind ^15^N-labeled holo-Fdx by NMR. We initially tested IscS_R39E/W45E, IscS_K101E/K105E, IscS_R220E/R223E/R225E, IscS_D346K/E347K, IscS_I314E/M315E, and IscS_E334S/R340S (Fig. 4). Of these mutants, only IscS_R220E/R223E/R225E did not bind to holo-Fdx: when titrated with IscS_R220E/R223E/R225E, no change was observed in the spectrum of holo-Fdx up to 4 molar eq as expected if mutation abolishes binding (Table 1). A similar behavior was observed for CyaY in the complex with IscS in agreement with CyaY and Fdx sharing the same binding site (19). Consistently, holo-Fdx, like CyaY, is an acidic protein (pI 4.49). The residues found to interact with IscS are all part of an acidic patch on holo-Fdx.

**TABLE 1:** **Summary of IscS mutant binding to holo-Fdx** ^1^H,^15^N-SOFAST HMQC spectra were used to observe [^15^N]holo-Fdx upon titration with IscS and IscS mutants.

IscS mutant	Observation in titration	Conclusion
IscS_WT	Signals disappear by 0.4 molecular eq uivalents	Binds
IscS_WT*^a^* ([^15^N/^2^H]Fdx)	Chemical shift perturbations observed, signals still observable at 0.4 molecular equivalents	Binds*^b^*
IscS_R39E/W45E	Signals disappear by 0.4 molecular equivalents	Binds
IscS_K101E/K105E	Signals disappear by 0.4 molecular equivalents	Binds
IscS_R112E/R116E*^c^*	Chemical shift perturbations observed, signals still observable at 0.6 molecular equivalents	Binds with weaker affinity
IscS_R220E/R223E/R225E	No chemical shift perturbations observed and signals still observable at 4 molecular equivalents	Does not bind
IscS_I314E/M315E	Signals disappear by 0.4 molecular equivalents	Binds
IscS_E334S/R340S	Signals disappear by 0.4 molecular equivalents	Binds
IscS_D346K/E347K	Signals disappear by 0.4 molecular equivalents	Binds

*^a^* [^15^N/^2^H]Holo-Fdx was used for the titration instead of ^15^N-holo-Fdx.

*^b^* Chemical shift perturbations were observed in addition to line broadening as a consequence of deuteration of holo-Fdx, which attenuated transverse relaxation and improved signal to noise.

*^c^* The IscS_R112E/R116E mutant was tested after the first round of holo-Fdx·IscS docking calculations by HADDOCK to validate the solution.

To quantify this hypothesis, holo-Fdx binding to the IscS mutants was measured by BLI. IscS_R39E/W45E, IscS_K101E/K105E, and IscS_E334S/R340S all had *K_d_* values indistinguishable from that of wild-type IscS, whereas no binding was observed with IscS_R220E/R223E/R225E (data not shown). Taken together, these results indicate that IscS residues Arg^220^, Arg^223^, and/or Arg^225^ are involved in the interaction with holo-Fdx.

##### Model Building and Experimental Validation of the Holo-Fdx·IscS Complex

Initial models of the holo-Fdx·IscS complex were generated using HADDOCK (28, 33). This method makes use of experimental data to guide molecular docking. IscS residues Arg^220^, Arg^223^, and Arg^225^ and holo-Fdx residues Gln^68^, Asp^70^, Asp^71^, Asp^74^, Lys^75^, Trp^77^, Leu^79^, Glu^80^, Pro^81^, and Glu^82^ were imposed as active AIRs with passive AIRs defined automatically by HADDOCK. Holo-Fdx residues Glu^69^, Met^72^, Leu^73^, Ala^76^, and Gly^78^ were excluded because they are solvent inaccessible. HADDOCK returned 190 structures distributed in seven clusters. The four lowest energy clusters differed by a slightly different orientation of Fdx in the same cavity centered on IscS residues Arg^220^, Arg^223^, and Arg^225^. Of these, the lowest energy cluster exclusively showed contacts between Glu^80^ and Glu^82^ of Fdx and Arg^112^ and Arg^116^ of IscS.

**FIGURE 5.**
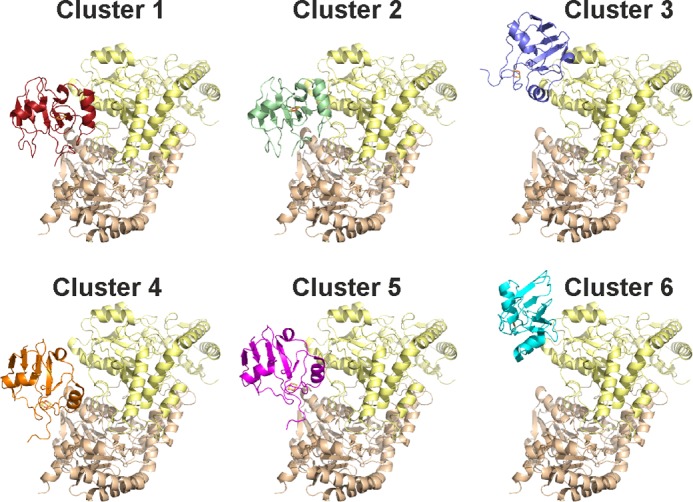
**Details on the six clusters obtained by HADDOCK calculations.** The corresponding energies are shown in Table 2.

To validate or reject this solution, we designed and tested an IscS_R112E/R116E. The chemical shift changes on Fdx are in the fast-exchange regime as opposed to slow-exchange for wild-type IscS. The peaks are still observable up to 0.6 molecular eq indicating that holo-Fdx binds IscS_R112E/R116E but with reduced affinity. We used this result to refine the input AIRs for the HADDOCK run obtaining six clusters (Fig. 5). Clusters 3 and 6 did not satisfy the AIRs for IscS Arg^112^ and Arg^116^ and so could be rejected. Clusters 1, 4, and 5 varied considerably with the position and rotational orientation of Fdx with respect to IscS. Clusters 4 and 5 were significantly less energetically favorable than Cluster 1 in terms of restraint violations (Table 2). Cluster 2 gave similar statistics as Cluster 1. The position and orientation of Fdx between Cluster 1 and Cluster 2 were similar, with root mean square deviation between the two lowest energy representatives of these clusters of 0.45 Å. Cluster 1 was taken as representative of the complex as this solution has better statistics.

**TABLE 2:** **Statistics for the lowest energy cluster solution for the IscS-holo-Fdx docking by HADDOCK**

	Cluster 1	Cluster 2	Cluster 3	Cluster 4	Cluster 5	Cluster 6
HADDOCK score	−147.7 ± 5.9	−144.9 ± 4.5	−101.7 ± 17.4	−92.6 ± 13.8	−88.4 ± 21.1	−35.4 ± 27.5
Cluster size	113	29	25	19	5	4
Root mean square deviation*^a^*	0.7 ± 0.5	1.1 ± 0.1	8.8 ± 0.3	3.9 ± 0.2	3.6 ± 0.3	9.7 ± 0.1
VdW energy*^b^*	−32.8 ± 3.8	−32.34.0	−37.5 ± 32.7	−34.9 ± 8.3	−22.6 ± 10.3	−12.8 ± 4.7
Electrostatic energy	−900.8 ± 16.5	−890.5 ± 44.5	−618.1 ± 32.7	−583.0 ± 82.3	−603.6 ± 87.6	−401.3 ± 113.0
Desolvation energy	65.2 ± 5.6	65.3 ± 9,2	58.5 ± 15.7	58.1 ± 7.4	54.7 ± 7.7	57.6 ± 8.2
Restraints violation energy	0.2 ± 0.23	1.2 ± 1.15	9.2 ± 12.38	8.0 ± 13.05	2.6 ± 1.38	0.7 ± 0.47
Buried surface area	2061.2 ± 57.8	2000.0 ± 186.2	1516.8 ± 103.2	1649.8 ± 164.6	1467.6 ± 290.2	982.2 ± 117.6
*Z*-score	−1.2	−1.1	0.0	0.2	0.4	1.7

*^a^* Root mean square deviation from the overall lowest energy structure.

*^b^* van de Waals energy.

**FIGURE 6.**
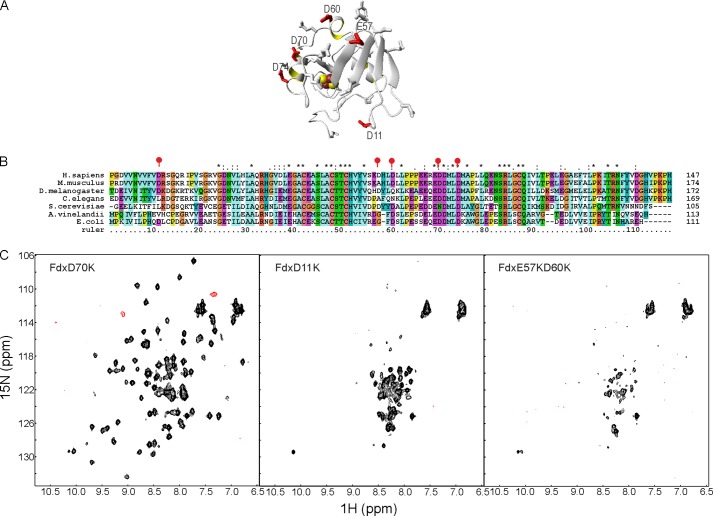
**Further model validation by designing *ad hoc* Fdx mutants.** *A,* mapping the mutations of Asp^11^, Glu^57^, Asp^60^, and Asp^70^ on the surface of the Fdx structure. The side chain of Asp^74^ is also shown. The cluster is represented as *red* and *yellow* spheres. *B,* multiple alignment of Fdx using the same species as selected in Fig. 4 for IscS. The mutated positions are marked by *red balloons*. Asp^70^ and Asp^74^ are completely conserved. *C,* titrations of ^15^N-labeled holo-Fdx_D70K (*left*), holo-Fdx_D11K (*middle*), and holo-Fdx_E57K/D60K (*right*) with IscS.

Using the predictive power of this holo-Fdx·IscS model, acidic to basic residue mutations were introduced in Fdx at residues Asp^70^, Asp^11^, and Glu^57^/Asp^60^ (Fig. 6*A*). Asp^11^, Glu^57^/Asp^60^, and Asp^70^ are in distinct exposed regions of holo-Fdx but only the latter is involved in interaction in our preliminary holo-Fdx/IscS model. The residues affected have different degrees of conservation (Fig. 6*B*). Binding between ^15^N-labeled holo-Fdx_D70K and IscS was not observed even up to 2.5 molar eq of IscS (Fig. 6*C*). Binding was instead retained as with wild-type holo-Fdx for ^15^N-labeled holo-Fdx_D11K and holo-Fdx_E57K/D60K. These results allow us to select a unique representative model of the holo-Fdx·IscS complex with excellent statistics (Table 2).

##### SAXS Analysis of Holo-Fdx·IscS Complex

**FIGURE 7.**
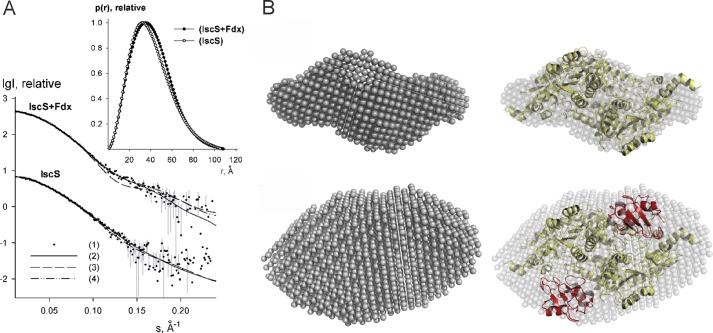
**SAXS analysis.** *A,* experimental SAXS data of the IscS and holo-Fdx·IscS binary complex are displayed as *dots* with *error bars* (*gray*), whereas *curves* computed from *ab initio* and crystallographic models (PDB 1P3W for IscS and HADDOCK model for holo-Fdx·IscS) are given as *solid* and *dashed lines*, respectively. The fit from OLIGOMER for the Iscs·Fdx binary complex with a χ value of 1.04 (yielding 80% of the complex and 20% of free components) is shown as *dash-dot lines*. The logarithm of the scattering intensity (*I*) is plotted as a function of the momentum transfer “s”. The successive curves are displaced down appropriately for better visualization. Distance distribution functions are shown in the insert. *B, ab initio* bead models of IscS (*top*) and holo-Fdx·IscS (*bottom*) (*gray semitransparent spheres*) superimposed with the crystal structure of IscS dimer and the NMR model of holo-Fdx·IscS binary complex. Crystallographic models of IscS and Fdx molecules are displayed as *yellow* and *red* C_α_ traces, respectively.

We acquired SAXS data for IscS alone and for the holo-Fdx·IscS complex in solution (Table 3 and Fig. 7*A*). The estimated apparent molecular mass (MM_exp_) and hydrated particle volume (*V_p_*) for IscS agrees with the presence of a dimer in accordance with previous SAXS results (19). The MM_exp_ and excluded volume of the holo-Fdx·IscS complex are clearly different from those of the isolated IscS and correspond to a stoichiometry of 2:2 for the binary complex. The overall parameters (*R_g_* = 31.0 Å, D_max_ = 112 Å) of holo-Fdx·IscS complex are close to those of the CyaY·IscS complex (*R_g_* = 31.1 Å, D_max_ = 109 Å) and differ significantly from those of the IscU·IscS complex (*R_g_* = 35.0 Å, D_max_ = 121 Å) excluding the possibility that Fdx binds on the periphery of the IscS dimer.

**TABLE 3:** **Overall structural parameters from SAXS data** MM, *R_g_*, D_max_, and *V_p_* denote the molecular mass, radius of gyration, maximum size, and excluded volume of the hydrated particle, respectively. Parameters without superscripts are experimental values; superscripts AB and XT refer to *ab initio* models and the crystal (IscS)/HADDOCK (holo-Fdx·IscS) structures, respectively. MM_calc_ is the theoretical molecular mass (MM) of IscS dimer (and 2:2 IscS·Fdx binary complex) computed from the protein sequence. χ is the discrepancy between experimental data and those computed from models.

Parameters	IscS	Holo-Fdx·IscS
MM_calc_ (kDa)	86	110
MM (kDa)	85 ± 10	105 ± 10
*R_g_* (Å)	30.7 ± 0.5	31.0 ± 0.5
D_max_ (Å)	110 ± 5	112 ± 5
*V_p_*, 10^3^ (Å^3^)	135 ± 10	155 ± 10
χ^AB^	0.99	1.06
χ^XT^	1.01	1.14

Multiple runs of the program DAMMIF (34), a fast version of DAMMIN (35) were used to produce average *ab initio* models. The shape envelope of isolated IscS overlaps well with the crystallographic dimeric structure (PDB 1P3W) (36) with a good fit to the data (Table 3 and Fig. 7*B*). The shape envelope of the holo-Fdx·IscS complex is more globular suggesting that the Fdx proteins are located close to the cavity (“pocket”) near the interface between the two IscS monomers. The HADDOCK model of the holo-Fdx·IscS complex can be well superimposed with the *ab initio* shape and yields a reasonably good fit to the data with a χ value of 1.14. Minor deviations at higher angles (*S* > 1.0 nm^−1^) can be explained by the presence of small populations (around 10%) of free components in solution (Fig. 7*A*) in agreement with the low affinity of the holo-Fdx·IscS complex.

##### Structural Analysis of the Holo-Fdx·IscS Model

**FIGURE 8.**
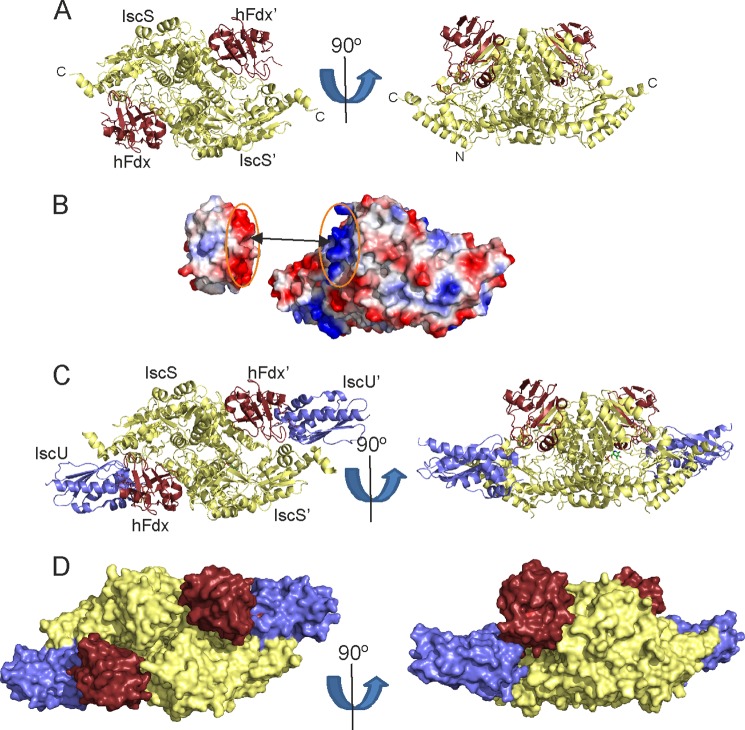
**Model of the holo-Fdx·IscS complex.** *A,* ribbon representations of the holo-Fdx·IscS HADDOCK model at orthogonal orientations. Fdx is shown in *brown*, the IscS dimer in *pale yellow. B,* surface electrostatic potentials of holo-Fdx and IscS (*red*, negative; *blue*, positive). The interface of the interaction is indicated by the *dotted ellipses. C,* ribbon representation of the model of the holo-Fdx·IscS·IscU complex IscU is indicated in *blue*. The model is shown at orthogonal orientations. *D,* the same as in *C* but using a surface representation.

Holo-Fdx binds in a cleft between the two IscS protomers (Fig. 8*A*) and covers a surface area of 2061.2 ± 57.8 Å^2^. The interaction is largely electrostatic (Fig. 8*B*), involving contacts between Asp^70^, Asp^71^, and Asp^74^ of holo-Fdx and Arg^220^, Arg^223^, and Arg^237^ of one IscS protomer. Glu^80^ and Glu^82^ of holo-Fdx interact with Arg^112^ and Arg^116^ of the second IscS protomer. The binding surface overlaps significantly with CyaY (19) in agreement with the competition experiments. Superimposition of the HADDOCK model of holo-Fdx·IscS with the crystal structure of the IscU·IscS complex (20) shows that IscS can accommodate both holo-Fdx and IscU in a hypothetical ternary holo-Fdx·IscS·IscU complex (Fig. 8*C*). In such a model, the [2Fe-2S] cluster of holo-Fdx is oriented between the active site cysteine loop of IscS and the cysteine ligands of IscU. The C terminus of holo-Fdx, which contains a histidine and a tyrosine, points toward the interface between IscS and IscU, and could be involved in electron transfer between the [2Fe-2S] cluster of holo-Fdx to the active site cysteine loop of IscS and/or the [2Fe-2S] cluster on IscU. This model provides the first structural insights into the holo-Fdx complex and its importance for cluster formation.

##### The Fdx·IscS Interaction Is Required for Fdx Function in Vivo

**FIGURE 9.**
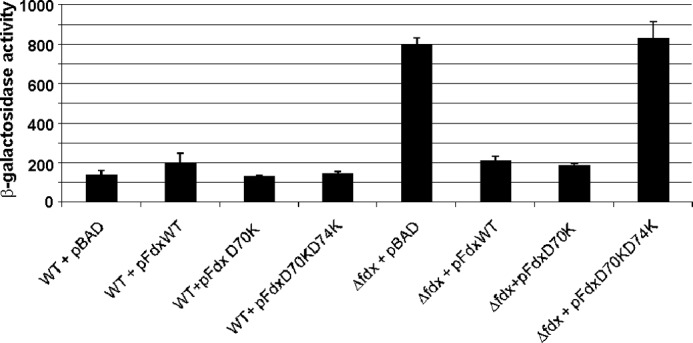
***In vivo* effect of mutated versions of the *fdx* gene in IscR maturation.** β-Galactosidase activity from wild-type (WT) and Δ*fdx* strains (Δ*fdx*) carrying the *P_iscR_*::*lacZ* fusion and transformed with pBAD, pFdxWT, pFdxD70K, or pFdxD70KD74K was measured in cells grown in LB supplemented with 50 μg/ml of ampicillin and 0.2% arabinose. Results shown are the mean of triplicate experiments.

Finally, we used an *in vivo* assay to test the importance of the residues involved in Fdx·IscS interaction in Fe-S biogenesis. We employed the *E. coli* Fe-S cluster-dependent transcriptional regulator IscR as reporter for Fe-S protein maturation and used an *E. coli* strain carrying the *lacZ* reporter gene fused to a gene whose expression is repressed by the Fe-S bound form of IscR, *iscR* (P*iscR*::*lacZ*) (31). As previously reported, introduction of *fdx* deletion leads to a defect in P*iscR* repression (Fig. 9) (37). This defect was recovered by complementing the Δ*fdx* strain with a wild-type pFdxWT plasmid and the pFdxD70K but not with the pFdxD70KD74K mutant (Fig. 9). These results indicate that mutation of these residues causes a defect in Fdx activity in Fe-S cluster biogenesis and highlight their importance *in vivo*.

## DISCUSSION

Identification of the machine that is responsible for iron-sulfur cluster biogenesis dates relatively recently (7). First described in prokaryotes, the components are highly conserved also in eukaryotes. Understanding how this machine works is an important task that relies on determining the full interactome in a time resolved way. Here, we have studied the interaction between Fdx and IscS, two essential components of the machine, using a hybrid methodology that combines NMR, SAXS, and mutagenesis. This approach has already proved successful to model the structure of the IscU·IscS complex, which is in excellent agreement with the x-ray structure (19). We obtained solid validation of our new results by NMR, SAXS, and extensive mutagenesis studies complemented by *in vivo* assay in *E. coli*.

We observe that IscS binds specifically holo-Fdx with a mainly electrostatic mechanism that involves complementary surfaces of opposite charge. Our results implicate a number of Fdx residues that may be required for the interaction with IscS. The fact that the FdxD70K single mutant is able to complement the Δ*fdx* strain suggests that Fdx Asp^74^ might be more important than Asp^70^ for the interaction with IscS and/or other binding partners. This is consistent with reports that demonstrate the importance of Asp^74^ in the primary interaction domain of human and bovine adrenodoxin (38–43).

Our results hold a number of important consequences not only for understanding the iron-sulfur cluster assembly machine but also for gaining new insights into multiple pathway regulation and disease. It has been proposed that *in vivo* Fdx may carry out the role of the reducing agent needed for accelerating reduction of S^0^ to S^2−^ in the transfer step from the persulfide of IscS to the [2Fe-2S] cluster on IscU and regenerating the reaction. This role is fulfilled *in vitro* by various reducing agents that are absent in the cell. It was also specifically suggested that “activation of cysteine desulfurases by accessory proteins can involve effects on either persulfide formation or its subsequent cleavage (or both)” (44). While the present work was under review, a report was published that fully confirms this hypothesis: it was shown that the presence of Fdx is sufficient for the IscS enzymatic reaction to occur without the need of other reducing agents (45). Our results suggest how this would happen mechanistically. The binding site of Fdx is close to the desulfurase active site and the flexible loop that transports the persulfide will have to go past Fdx on its way to IscU. Electron transport could be mediated by Tyr^103^ and/or His^107^ of Fdx, which could assist, through a relay mechanism, their transfer to IscU. An alternative but not mutually exclusive role for Fdx was suggested to be an involvement in reductive coupling of two [2Fe-2S]^2+^ clusters to [4Fe-4S]^2+^ clusters (9, 10, 46). Although certainly plausible and supported by experimental data, we can exclude that this function is carried out when Fdx is in the bound state with IscS because IscU can only bind IscS as a monomer. This reaction is therefore possible only when cluster-loaded IscU detaches from IscS.

Some open questions remain that will need further investigations. We observe that Fdx competes for the same binding site that accommodates CyaY, whereas leaving the interaction with IscU unchanged. In addition, the Fdx binding site overlaps not only with CyaY but also with at least two other proteins, YfhJ (IscX), part of the ISC operon, and TusA (20). IscS forms additional interactions with Thil and MoeB/MoeD (47, 48), although it is not known precisely where they bind. The latter three proteins are not in the operon and are implicated in tRNA modification and molybdenum cofactor biosynthesis. This complex network of mutually exclusive interactions poses the intriguing problem of which component is bound at any given time and thus how the different metabolic pathways are regulated. We are confident that our work will help shed light on the process and inspire further studies that are able to elucidate this complex problem.

## Footnotes

2 The abbreviations used are:

Fdx: ferredoxin
EPR: electron paramagnetic resonance
SAXS: small angle x-ray scattering
HMQC: heteronuclear multiple quantum coherence
BLI: biolayer interferometry
ISC: iron-sulfur cluster
AIR: ambiguous interaction restraint.
